# An Adapted Hybrid of Open and Laparoscopic Techniques for Ventral Hernia Repair in a Resource-Constrained Setting

**DOI:** 10.7759/cureus.81095

**Published:** 2025-03-24

**Authors:** Arvin Khamajeet, Ahmed Diab, Samantha Marchant, Heather Bougard

**Affiliations:** 1 General Surgery, University of Cape Town, Cape Town, ZAF; 2 General Surgery, New Somerset Hospital, Cape Town, ZAF; 3 General Surgery, University of Cape Town/New Somerset Hospital, Cape Town, ZAF

**Keywords:** hybrid ventral hernia repair, ipom plus, laparoscopic ventral hernia repair, open ventral hernia repair, ventral hernia repair

## Abstract

Ventral hernias are common surgical conditions managed through various techniques, including open, laparoscopic, and hybrid approaches. Laparoscopic repairs offer advantages such as reduced postoperative pain and faster recovery but carry an increased risk of bowel injury. Of all the repairs, retrorectus repair has demonstrated superior long-term outcomes. In resource-limited settings, adapting existing techniques, with consideration of cost, is essential to enhance patient safety and surgical efficiency.

This report presents the case of a 65-year-old female, known hypertensive with a BMI of 33, presenting to a regional hospital in South Africa, with a symptomatic ventral hernia measuring 51 mm × 36 mm. The hernia contained portions of the transverse colon and omentum. Due to the symptomatic nature and risk of complications, surgical intervention was indicated. A hybrid technique combining open and laparoscopic methods was employed, accessing the retrorectus plane via a small incision, followed by gel port placement to facilitate laparoscopic dissection and mesh placement. The patient had an uneventful recovery, was discharged on postoperative day two, and experienced no complications on follow-ups.

This case highlights a cost-effective, minimally invasive adaptation of retrorectus repair suitable for resource-constrained settings. The technique minimizes surgical trauma and enhances cosmetic outcomes while maintaining the benefits of minimally invasive surgery. Further studies are needed to evaluate long-term outcomes and broader applicability.

## Introduction

Ventral hernias can be either congenital or acquired. The incidence of incisional ventral hernias ranges from 6% to 81% and may be higher depending on the study [1]. Repair options include primary repair, mesh repair, and laparoscopic versus open surgery. Primary repair can be performed in patients with a defect size of less than 2 cm and no risk factors for recurrence, such as obesity, recurrent hernias, or diastasis [2]. Mesh repair is indicated for defects larger than 2 cm or in the presence of risk factors for recurrence [2]. Two of the most commonly used methods for ventral hernia repair are laparoscopic intraperitoneal onlay mesh (IPOM) repair and retrorectus repair, which can be performed using either open or minimally invasive techniques. Kockerling et al. demonstrated that retrorectus repair was superior to other types of open repairs [3]. Chalabi et al. and Dietz et al. both found that laparoscopic and retrorectus repairs had similar recurrence rates [4,5]. However, laparoscopic surgery appears to be associated with fewer complications [3]. A recent study has also shown the benefits of minimally invasive retrorectus repair compared to open retrorectus repair [6]. This study aims to describe a hybrid technique for retrorectus repair in a resource-constrained setting to reduce the invasiveness of the procedure.

## Case presentation

A 65-year-old female with a history of hypertension, well-controlled on a single antihypertensive agent, and a BMI of 33, presented with a de novo midline ventral hernia. She had been aware of the hernia for the past 10 years; however, it had recently become symptomatic, with the onset of abdominal pain persisting for the past two to three weeks. Her past surgical history was notable only for a bilateral tubal ligation performed 33 years ago.

**Figure 1:**
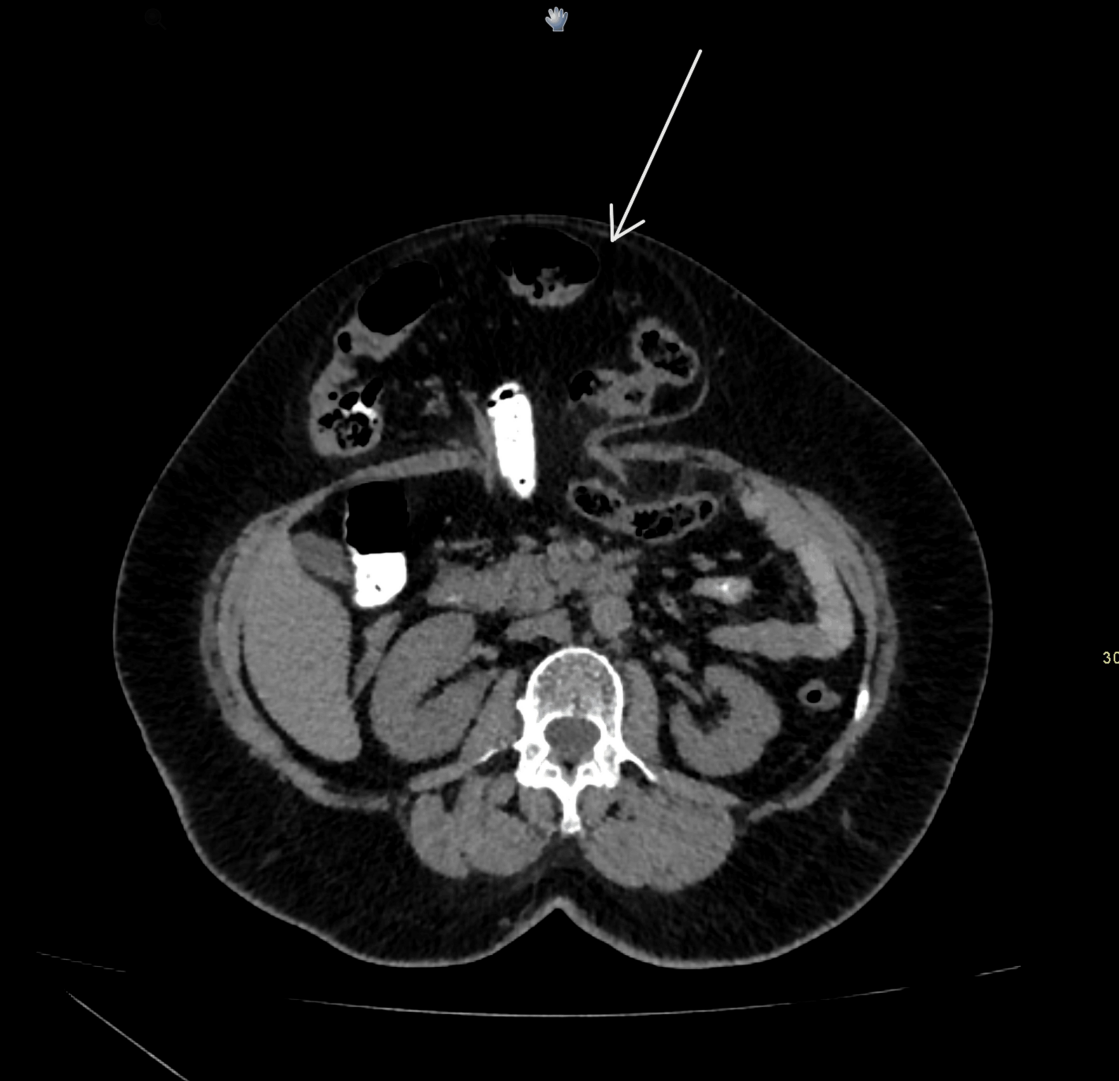
Non-contrast CT with oral contrast showing an axial view with bowel in the hernial sac (arrow)

**Figure 2:**
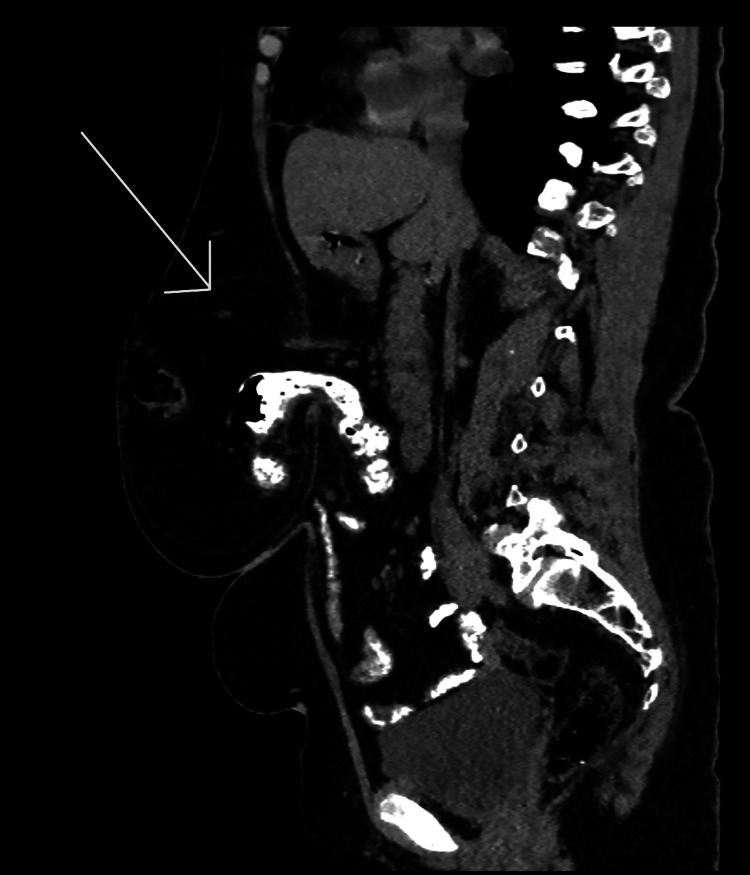
Sagittal view of a non-contrast CT scan with oral contrast showing bowel in the hernial sac (arrow)

A CT scan (Figures 1, 2) revealed a large midline anterior abdominal wall defect measuring 51 mm by 36 mm, with herniation of parts of the transverse colon and omentum. Given the symptomatic nature of the hernia and the presence of abdominal wall contents within the defect, surgical intervention was deemed necessary to prevent further complications.

The patient underwent surgery, and the hybrid technique we used is hereby described. We chose a hybrid technique because we felt it was a complex repair due to the presence of a bowel in a long-standing hernia. Repairing the hernia via a small incision will reduce complications such as chronic pain and recurrence. We adapted the MILOS procedure to our resource constraints by using a gel port for the camera and laparoscopic port insertion.

Technique

**Figure 3:**
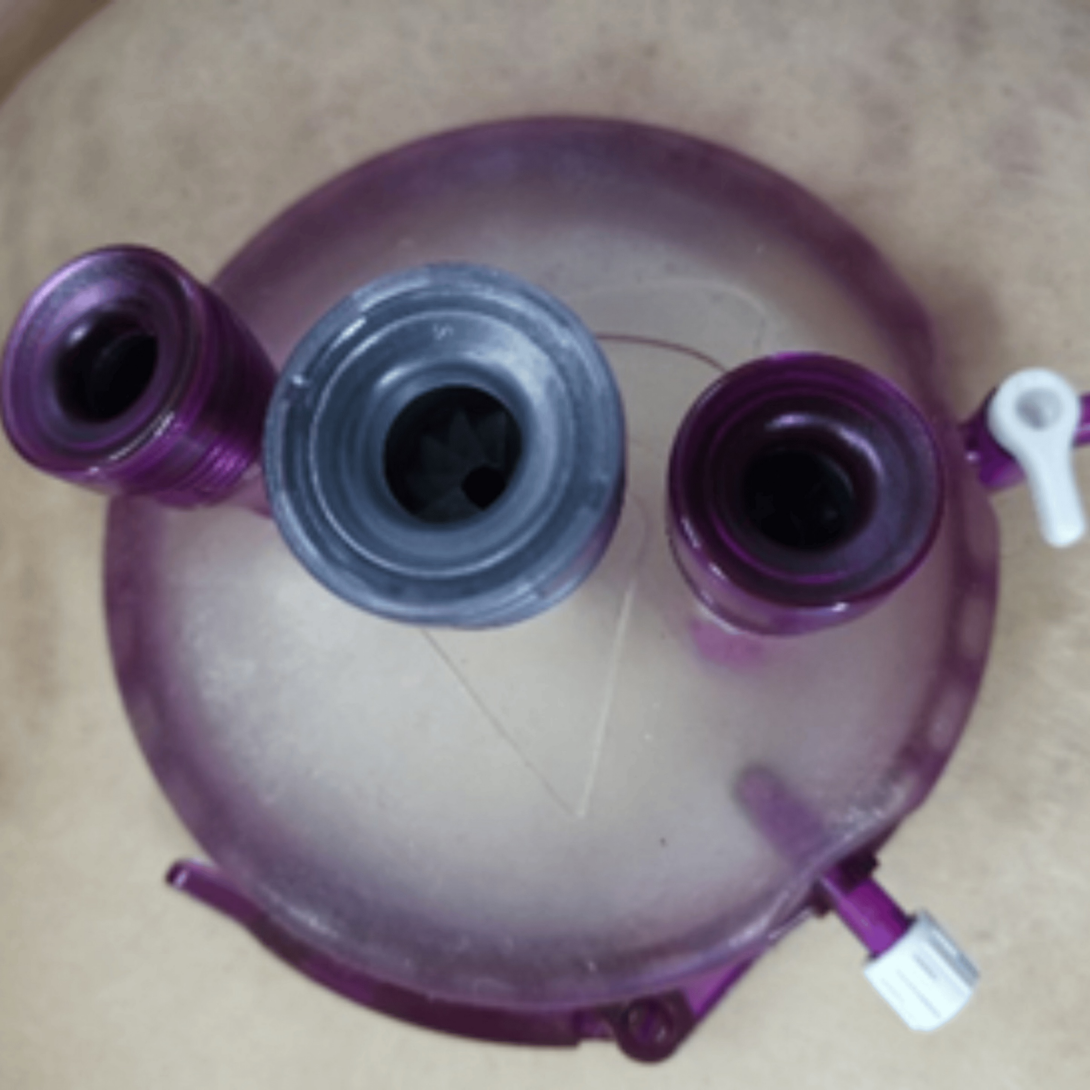
A single gel port was placed over the wound protector after the reduction of hernial contents and partial mobilization of the retrorectus space, following the closure of the posterior sheath

The surgery was performed under general anesthesia. The patient was positioned in the supine position, and a vertical incision, approximately 8 cm in length, was made over the ventral hernia site. The skin incision was deepened until the hernial sac was reached. The hernial sac was opened, and its contents were carefully reduced back into the abdomen. The posterior sheath was then closed with Vicryl 2/0 sutures, and a wound protector for the gel port was applied following the open mobilization of the retrorectus space to the extent possible (Figure 3).

**Figure 4:**
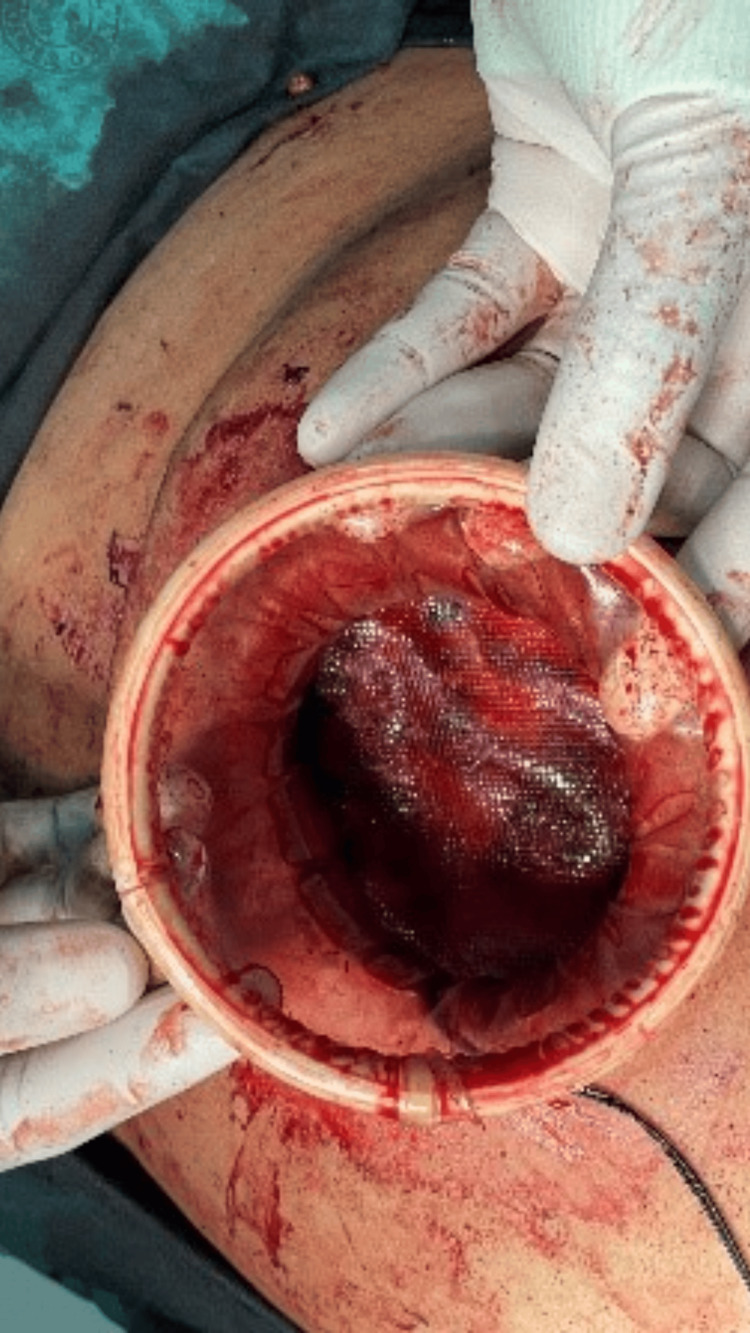
Application of mesh following the development of the retrorectus space via laparoscopy

**Figure 5:**
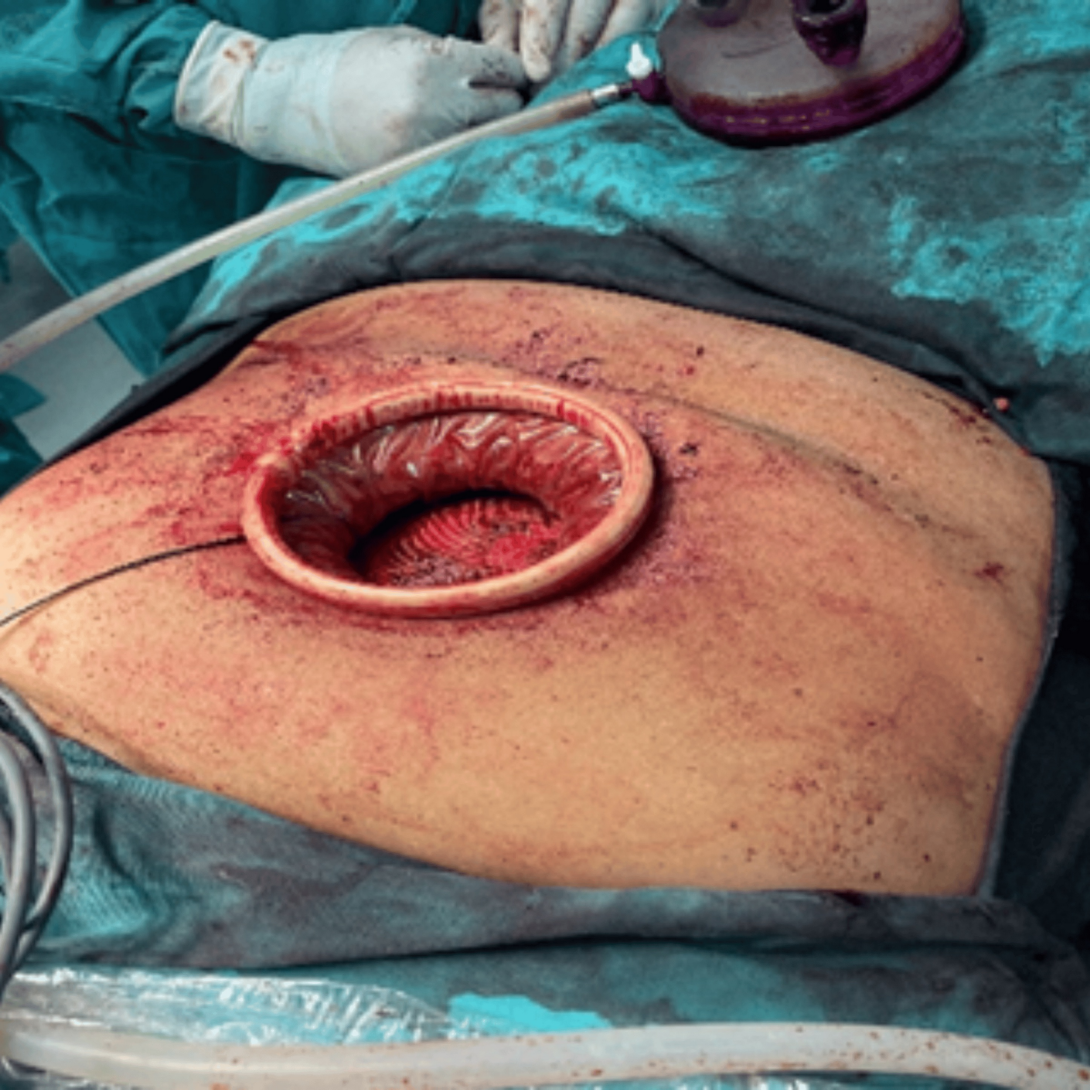
View of the mesh after placement

**Figure 6:**
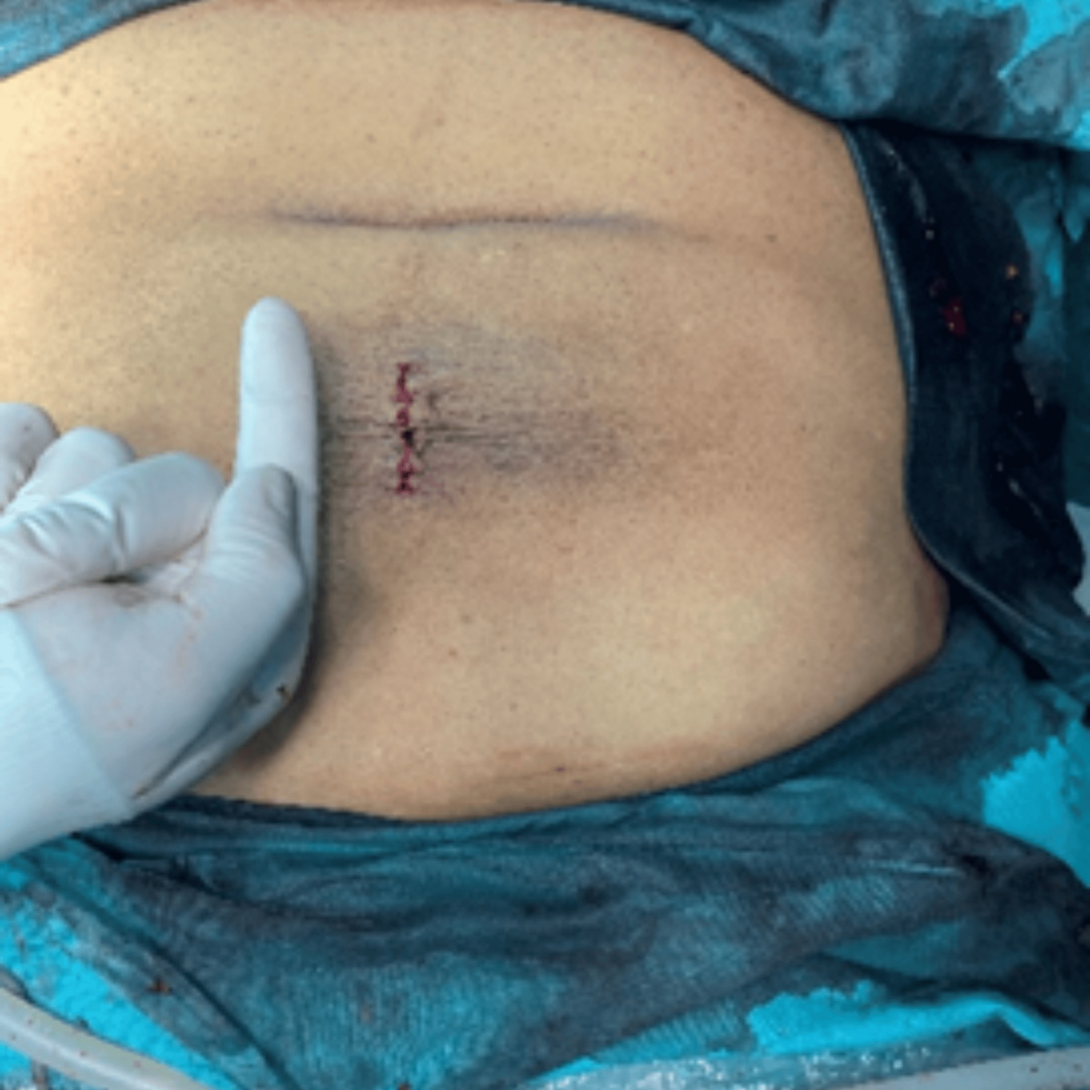
Closure of the skin following mesh placement

A 30-degree camera was introduced through the central port, allowing laparoscopic instruments to be used to further develop the retrorectus plane. Finally, an Optilene polypropylene mesh was inserted in an open manner after the precise measurement of the retrorectus space to ensure adequate coverage (Figure 4 and Figure 5), with a minimum of 5 cm overlap. No tacking or sutures were used as the mesh was placed in the retrorectus space. The anterior sheath was closed with Nylon 1/0 sutures, and the skin was closed with absorbable Monocryl 3/0 sutures (Figure 6). The duration of the surgery was 2 hours, and blood loss was minimal, estimated at less than 50 mL.

Intraoperative and postoperative follow-ups

The patient had no intraoperative or early postoperative complications and was discharged on postoperative day 2 in stable condition. On follow-up, the patient had no recurrence of hernia, no late complications, and was managing all daily activities without any discomfort.

## Discussion

Ventral hernias represent a commonly encountered condition in the surgical field. A study by Beadles et al. revealed an incidence of emergency hernia operations for umbilical and incisional hernias at 13.8%. It is noteworthy that emergency surgery is associated with increased morbidity and mortality, highlighting the need to repair these hernias in the elective setting. Methods for repairing these hernias vary, ranging from open surgery to minimally invasive to hybrid techniques. Forbes et al. demonstrated equal or superior benefits of laparoscopic repair compared to open retrorectus repair [7]. However, Sauerland et al. showed that laparoscopic hernia repairs have an increased rate of bowel injuries compared with open surgery, a finding supported by another study conducted by Le Blanc et al., which found a 1.7% rate of enterotomy and an associated mortality of 2.8% if diagnosed [8,9]. Additionally, laparoscopic repair is not recommended for hernial defects greater than 10 cm [10]. This implies that reducing the risk of bowel contact could potentially decrease the associated morbidity and mortality. Laparoscopic transperitoneal sublay mesh repair has been proposed as a technique for retrorectus repair and has shown favorable benefits, including early discharge and lower costs due to the reduced need for tacker devices [11]. This technique also minimizes contact between the mesh and the bowel, potentially reducing the rate of fistulas and adhesions. The Milos technique, which stands for minimally or less open sublay technique, is a hybrid approach previously described in the hernia literature with good results compared to the IPOM or open approach, showing fewer complications such as chronic pain and recurrences [12]. The technique is similar to ours, except for a specifically designed endotorch used by the unit that developed the technique [12]. Our unit has bypassed the use of this specially designed light torch by employing a gel port and conventional laparoscopic equipment for dissection. Our hybrid technique enables safe mobilization of the retrorectus space with a smaller incision, providing a better cosmetic outcome from the outset and causing less interference with the stronger, normal linea alba compared to conventional open techniques. The repurposing of a single-port, single-use gel port device introduces this advanced technique, along with the documented robust outcomes of the eMILOS procedure, into the armamentarium of the hernia surgeon. It also allows the performance of this procedure using existing gel port equipment and a wound protector.

## Conclusions

Ventral hernias represent a common surgical problem, with laparoscopic hernia repairs and retrorectus repairs having more favorable outcomes, as laparoscopy is associated with many benefits over the open procedure. It is noted, however, that retrorectus repairs are favored for hernias above 10 cm. Our technique represents a lower-cost hybrid approach that may be used in resource-constrained settings to reduce complications.
